# Microbial proliferation deteriorates the corrosion inhibition capability, lubricity, and stability of cutting fluid

**DOI:** 10.3389/fmicb.2025.1522265

**Published:** 2025-02-11

**Authors:** Yuanyuan Shen, Wenkai Zhang, Lili Wu, Yaohua Dong, Guoqiang Guo, Lihua Dong, Zhangwei Guo

**Affiliations:** ^1^Institute of Marine Materials Science and Engineering, College of Ocean Science and Engineering, Shanghai Maritime University, Shanghai, China; ^2^Shanghai Spaceflight Precision Machinery Institute, Shanghai, China

**Keywords:** microbial reproduction, deterioration of cutting fluid performance, corrosion inhibition capability, lubricity, stability, microbial metabolism

## Abstract

Cutting fluid is a type of fluid used in the metal-cutting process. It is prone to microbial growth during use, which can lead to the deterioration of its various useful properties; however, the mechanism underlying this deterioration remains unclear. This study analyzed the microbial diversity of field-sampled cutting fluids, and those with higher levels of diversity were used to inoculate other fluid samples in order to further study the effects of microbial growth on the properties of cutting fluids. The results show that the surface of cutting fluid sampled from the tank of a machining tool tank contained predominantly aerobic bacteria, while the bottom mainly harbored anaerobic and facultative microorganisms, with *Yarrowia lipolytica* representing the dominant fungus. Some obligate anaerobic bacteria were also present in the cutting fluid. Organic acids secreted by anaerobic microbial activity reduced the pH of the cutting fluid, as well as its resistance to corrosion. The metabolic activity of the aerobic microorganisms also consumed certain key components of the cutting fluid, which ultimately further lowered its pH and resistance to corrosion. Moreover, the number of fungi increased significantly during the later stages of the experiment. The rolling and bridging action of the resulting fungal mycelium caused flocculation of the effective components in the cutting fluid, resulting in reduced lubricity and poor stability. This study provides a theoretical basis for developing more effective measures to inhibit microbial growth and delay the deterioration of cutting fluid, thereby helping to improve the technical quality of the metal-cutting industry.

## 1 Introduction

Cutting fluid (i.e., coolant) is a type of industrial liquid used for cooling, lubricating, cleaning, and preventing corrosion during the metal-cutting process. It comprises and is synthesized using a variety of functional chemical additives (Osama et al., 2017; Choudhury and Muaz, 2020; Chen et al., 2024). In recent years, as cutting speeds have increased in this industry, greater requirements are being placed concerning the safety and cooling properties of cutting fluid, leading to the gradual replacement of traditional oil-based cutting fluids with water-based ones. These water-based cutting fluids mainly contain water and mineral oil, along with anti-corrosion agents, defoamers, lubricants, and other additives with different chemical structures that ensure their performance and stability (Simpson et al., 2000; Lillienberg et al., 2008; Lillienberg et al., 2010). These additives are organic, and thus can represent nutritional sources for certain microorganisms (Trafny, 2013).

Microorganisms can enter cutting fluids in various ways. For example, tap water—which is often used to dilute concentrated cutting fluid—can represent a significant source of microbial contamination. Residues from used cutting fluids often remain even after pipelines or the dead spaces of machines have been cleaned, thus representing another source of microbial contamination when new fluids are added to such cutting systems. Microorganisms also enter cutting fluids through the equipment, or be inoculated by the equipment operators. Fresh cutting fluids are also susceptible to contamination by microorganisms from dust particles or aerosols. Such microorganisms not only threaten the health of workers in the industry, but also lead to spoiling of the cutting fluid, which can produce foul odors and affect its physicochemical properties (Liu et al., 2010; Schwarz et al., 2015). This, in turn, can lead to deterioration of the quality of the workpiece and reduce the lifespan of the tool (Rakić and Rakić, 2002).

Studies have shown that microorganisms are highly capable of growth in water-based cutting fluids from machining plants, leading to environmental malodor, fluid discoloration, and increased acidity (Gast et al., 2001; Grijalbo et al., 2015). After isolating fungi and bacteria from cutting fluids, Elansky et al. investigated their growth in cutting fluid, and hypothesized that microorganisms could be used to treat metal-working fluid waste through biodegradation (Elansky et al., 2022). Hill found that microbial growth consumes certain components of cutting fluid and increases the diameter of the droplets formed by its dispersion, which in turn leads to decreased performance that affects the machining accuracy of workpiece surfaces (Hill, 1992). Rabenstein et al. studied the microbial community in water-based metal-working fluids and the microbial degradation of their active ingredients, ultimately demonstrating that the active ingredients were not consumed simultaneously, but rather in a sequential order (Rabenstein et al., 2009). Zhang et al. investigated the effect of corrosion caused by microorganisms on cemented carbide (WC-30Co) cutting tools in an oil–water emulsion (Zhang et al., 2015). They found that preferential cobalt loss determined the corrosion failure in biodegraded emulsions, and that the sulfate-reducing bacterium *Citrobacter* sp. made the emulsion even more corrosive to the hard metal. To date, the safety hazards and economic losses caused by microorganisms have attracted significant attention in the fields of marine engineering, pipeline transportation, construction engineering, and nuclear power (Yazdi et al., 2022; Lu et al., 2023; Sun et al., 2023; Li et al., 2024). Conversely, microbial growth in cutting fluid, and the related deterioration of its performance, as well as the environmental pollution caused by the resultant waste (Sankaranarayanan et al., 2021; Korkmaz et al., 2023), has not yet attracted sufficient attention. This has resulted in a scarcity of research concerning the underlying mechanisms of its degradation.

The main objectives of this study were to: (1) examine the diversity and characteristics of the microbial communities in industrial cutting fluid; (2) study the effects of microbial growth and metabolism on the performance of industrial cutting fluid, based on parameters such as pH, anticorrosion, lubrication, and stability, under laboratory conditions; and (3) analyze the mechanism underlying the microbially-induced deterioration of industrial cutting fluid. The theoretical data provided herein will hopefully be of great significance to preventing microbial corrosion in industrial cutting fluid, as well as for maintaining and extending the life of cutting fluids used in metal-processing operations.

## 2 Materials and methods

### 2.1 Microbial sampling

Cutting fluid samples (labeled “CF”), were collected from the surface and bottom of four equally-sized machine tool tanks (labeled 1, 2, 3, and 4), that had been filled with the same cutting fluid, used to cut the same metal, and received equal use at a research institute for aerospace materials in Shanghai, China. All of the cutting fluids had been in use for 4 months prior to the study, during which they had blackened and begun emitting a foul odor. Samples (50 mL) of each fluid were centrifuged in a high-speed refrigerated centrifuge (Multifuge X1R, Thermo Fisher Scientific, Waltham, MA, United States) at 44,720 × g and 4°C for 10 min. The supernatants were discarded, and the pellets were resuspended and diluted in physiological buffered saline (PBS), after which the resultant solutions were centrifuged again under the same conditions. The final pellets were then analyzed for biodiversity via high-throughput sequencing.

### 2.2 Microbial community analysis

#### 2.2.1 DNA extraction and quality assessment

Microbial DNA from the cutting fluid samples was extracted using a FastDNA Spin Kit for Soil (MP Biomedicals LLC, Irvine, SC, United States), after which the concentration and purity of the extracted DNA were measured using a NanoDrop 2000 fluorometer (Thermo Fisher Scientific, Waltham, MA, United States).

#### 2.2.2 Polymerase chain reaction amplification

The V3–V4 hypervariable region of the bacterial 16S rRNA gene was amplified using the universal primers 338F (5′-ACTCCTACGGGAGGCAGCAG-3′) and 806R (5′-GGACTACNNGGGTATCTAAT-3′). The fungal ITS2 region was amplified using the universal primer sets ITS1 (5′-CTTGGTCATTTAGAGGAAGTAA-3′) and ITS2 (5′-TGCGTTCTTCATCGATGC-3′) (Abarenkov et al., 2010). Both the 16S rRNA and ITS rRNA amplicons were generated using the same protocol. The PCR mixtures comprised 5× buffer (10 μL), forward primer (1 μL), reverse primer (1 μL), deoxy-ribonucleoside triphosphates (dNTPs) (10 μL), double-distilled water (ddH2O), Phusion high-fidelity DNA polymerase (0.5 μL, New England Biolabs, Inc., Ipswich, MA, United States), and template DNA (30 ng), with total reaction volumes of 50 μL. The cycling parameters were: 94°C for 2 min; followed by 24 cycles of 94°C for 30 s, 55°C for 30 s, and 72°C for 30 s; before a final extension at 72°C for 5 min (Rognes et al., 2016). The generated PCR products were recovered via 2% agarose gel electrophoresis before being purified using an AxyPrep DNA Gel Extraction Kit (Axygen Biosciences, Silicon Valley, CA, United States). A Quantus™ fluorometer (Promega Corporation, Madison, WI, United States) was used for nucleic acid quantification of purified PCR products. Sequencing was then performed on an Illumina MiSeq PE3000 platform (Illumina, Inc., San Diego, CA, United States) at Shanghai Meiji Biomedical Technology Co., Ltd., China (Zhang et al., 2014). The data presented in the study are deposited in the National Center for Biotechnology Information (NCBI) repository, accession number SRP554559: PRJNA1204126.

#### 2.2.3 Bioinformatic analysis

The raw fastq files were demultiplexed based on the barcode. PE reads for all samples were run through Trimmomatic (version 0.35) to remove low quality base pairs using these parameters (SLIDINGWINDOW: 50:20; MINLEN: 50) (Pruesse et al., 2007). Trimmed reads were then cut primer and adaptors using cutadapt (version:1.16). And then further merged using FLASH program (version 1.2.11) with default parameters. The low quality contigs were removed based on screen.seqs command using the following filtering parameters, maxambig = 0, minlength = 200, maxlength =485, maxhomop = 8.

The 16S sequences were analyzed using a combination of software mothur (version 1.33.3),UPARSE (version v8.1.1756), and R (version 3.6.3). The demultiplexed reads were clustered at 97% sequence identity into operational taxonomic units (OTUs) and the chimera was removed and the singleton OTUs were deleted using the UPARSE pipeline (Edgar et al., 2011; Edgar, 2013). The OTU representative sequences were assignment for taxonomy against Silva 128 database with confidence score ≥ 0.6 by the classify.seqs command in mothur. As for the ITS taxonomic analysis, the UNITE database was used.

For the alpha-diversity analysis, Shannon, simpson, Chao1, ACE index and rarefaction curves were calculated and plotted by R. For the beta-diversity metrics, the weighted and unweighted UniFrac distance matrix were calculated and visualized with Principal Coordinate Analysis (PCoA) by ape package in R, NMDS by vegan package in R and tree by dendextend package in R (Harmon-Smith et al., 2010). The bray curits and jaccard metrics were calculated by vegan package in R and visualized also by R the same as Unifrac analysis.

### 2.3 Experimental solutions

In order to better analyze the effects of microorganisms on cutting fluid performance, the cutting fluid formulation was simplified and its fungicide was removed for this experiment. The fluid’s composition therefore comprised: 4.9% nonylphenol polyoxyethylene ether, 7.8% triethanolamine, 9.6% fatty alcohol polyoxyethylene ether, 9.6% dehydrated sorbitol fatty acid ester (i.e., Span), 9.6% polyethylene glycol dioleate, 50.3% mineral oil, 6% tall oil, 0.2% organic silicon, and 2% organic phosphate ester (all proportions in w/w). Before the formal experiments, the simplified cutting fluid was diluted to a volume fraction of 5% wt (the concentration recommended by the supplier) with deionized water, and dubbed “SCF.” This fluid contained sufficient organic components to supply ample nutrients for microbial growth, so it was not further supplemented with any additional nutrients. The total volume of this solution was 1 L.

### 2.4 Microbial growth curve

To ensure high microbial diversity, CF samples with higher levels of biodiversity were selected as sources of the microbial strains. Appropriate amounts of spoiled CF were inoculated into 100 mL aliquots of sterilized SCF solution, to initial bacterial concentrations of ~10^6^ CFU/mL. Since the cutting fluid was milky-white, it was difficult to accurately determine its microbial concentration using the absorbance method. Therefore, to plot the growth curve of the microorganisms in the fluid, microbial concentrations at different incubation times were quantified via the plate-count method. Three replicate solutions were prepared to increase the experiment’s accuracy. The main components of the solid medium used in the plates were: beef extract (5 g/L), peptone (5 g/L), triethanolamine borate and its derivatives (100 g/L), oleic acid (5 g/L), methanol (3 g/L), organophosphate ester and its derivatives (3 g/L), and agar (15 g/L) (Yin et al., 2019). The bacterial solution was appropriately diluted via step-wise dilution. Aliquots of the diluted microbial suspension (1 mL) were taken using sterile pipettes and inoculated onto the solid medium in Petri dishes, before being evenly spread using a sterilized glass scraper. The plates were then left face-up on the sterile experimental benchtop for 30 min to allow the solution to penetrate the medium, before being inverted for culturing in a controlled-temperature incubator at 37°C. The plates were removed at different time points, and Scan 300 colony-counters (Interscience, Puy Capel, Cantal Province, France) were used to count the resultant colonies.

### 2.5 Testing of physical and chemical properties

The SCF solution was packed in two 1 L conical flasks that were plugged with cotton plugs, sterilized in an autoclave at 121°C for 20 min, then air-cooled in a biosafety cabinet. One of the conical flasks was inoculated with 200 μL of a CF sample with high microbial diversity, which was dubbed “Test-SCF.” The other conical flask was not inoculated, to serve as a control, and dubbed “Control-SCF.” These two conical bottles were incubated at 38°C for static culturing. After 1 month, samples from the middle regions of the solutions within the flasks were taken so that the compositions of their bacterial and fungal communities could be analyzed.

#### 2.5.1 Cutting fluid pH and redox potential

To study the effect of microbial growth on pH, he pH levels of the two SCF solutions were measured at 7, 14, 21, and 28 days using an FE20 pH meter (Mettler-Toledo International Inc., Zurich, Switzerland). The redox potential (ORP) of two solutions was measured at various stages throughout the experiment using an SX712 ORP meter (Shanghai San-Xin Instrumentation, Inc., Shanghai, China).

#### 2.5.2 Cutting fluid corrosion-inhibiting properties

The corrosion inhibition ability of the cutting fluid was evaluated using the iron filings method. A section of qualitative filter paper was placed into a Petri dish, after which 2 g of iron filings were uniformly spread over its surface, with the weight confirmed using a precision balance. A 1 mL sample of SCF was then added to saturate the iron filings, and the dish was sealed using its lid. After incubating at room temperature for 2 h, the filter paper was removed and the iron filings were washed with running water. The number of rust spots was then counted, once the filter paper had dried completely.

Given the use of both onsite-sampled CF and the laboratory-prepared SCF, both needed to be analyzed regarding the impact of microbial contamination on their abilities to inhibit corrosion. Electrochemical analyses, including measurements of open circuit potential (OCP) and polarization curves, were conducted using samples of 7,050 aluminum alloy (Shanghai Hanglv Aviation Materials Co., Ltd., Shanghai, China) that were exposed to spoiled cutting fluids for varying periods. The aluminum alloy, sourced from an aerospace materials research institute in Shanghai, comprised 0.04% Cr, 0.12% Zr, 6.2% Zn, 0.11% Si, 0.08% Fe, 0.06% Mn, 2.3% Mg, 0.05% Ti, and 0.04% Cu, with the remaining composition being aluminum. Prior to testing, the alloy was sectioned into 10 × 10 × 2 mm samples that were sequentially polished under wet conditions with 800 grit silicon carbide paper, ultrasonically cleaned in anhydrous ethanol for 10 min, and air-dried. Lengths of copper wire were then soldered to the reverse sides of each sample. The soldered faces and remaining samples were encapsulated in epoxy resin, leaving only 10 × 10 mm working surfaces exposed on each. These working surfaces were further polished using 800 grit water-resistant sandpaper, sequentially cleaned with anhydrous ethanol and deionized water, and dried before being used for electrochemical evaluation.

Electrochemical evaluation was performed using a CHI 660 electrochemical workstation (Shanghai Chenhua Instrument Co., Ltd., Shanghai, China). The experimental configuration used a three-electrode system comprising a saturated calomel electrode (SCE) as the reference, a platinum plate (10 × 10 mm) as the counter electrode, and aluminum alloy samples immersed in experimental cutting fluid samples as the working electrodes. The sterilized SCF comprised the electrolyte used to assess the electrochemical behavior of the working electrode. After being immersed for varying lengths of time, samples were extracted from the conical flasks and tested in an electrochemical testing system. To ensure the system’s stability prior to the measurements, all of the samples were equilibrated at OCP for 30 min in a sterile environment at ambient temperature.

Polarization curve tests were carried out at a scan rate of 5 mV/s and range of −1 to +1 V relative to the stabilized OCP. The resultant polarization curve data were analyzed and fitted using the electrochemical workstation’s integrated analysis software.

#### 2.5.3 Cutting fluid lubricity

Lubricity is commonly quantified by measuring tapping torque, with higher values signifying better lubricity. As the experiment progressed, the effects of microbial activity on the tapping torque values of the cutting fluid samples were quantified using a Labtap G8 threading torque meter (Microtap, Taufkirchen, BY, Germany). These measurements used aluminum alloy 7,075 for the standard extruded-hole test block, with a hole diameter and depth of 3.7 mm and 20 mm, respectively. A syringe was used to inject the solutions into the holes until they were fully filled. The tap used for extrusion molding had a diameter of 3.64 mm. The tests were conducted at a set rotational speed of 1,500 rpm, an applied torque of 400 N·cm, and a tapping depth of 12 cm.

#### 2.5.4 Cutting fluid stability

The stability of industrial cutting fluid is primarily assessed via two methods: oil droplet morphology analysis and zeta potential measurement. In general, smaller molecules or dispersed particles within the fluid correspond to greater absolute zeta potential values (positive or negative), signifying higher stability.

The methodology used to assess the oil droplet morphology in the cutting fluid samples involved staining with Oil Red O. A 1 g aliquot of Oil Red O was dissolved in 2 mL of anhydrous ethanol and mixed thoroughly to prepare the dye solution. A 100 μL aliquot of this dye was then added to 1 mL of the fluid being analyzed, followed by vigorous shaking to achieve uniform staining. The morphologies of the resultant oil droplets were then examined using a DM500 optical microscope (Leica, Wetzlar, HE, Germany).

Zeta potential was measured using a Litesizer 500 nanoscale particle analyzer (Anton Paar GmbH, Graz, ST, Austria) at various time points over the course of the experiment. Measurements were performed in an omega-shaped sample cell maintained at a target temperature of 25°C, with an equilibration time of 1 min. The Smoluchowski approximation was used, with Henry’s function set at 1.5. The adjustment mode was automatic, the running mode was manual (100 runs), and water was used as the solvent.

### 2.6 Microbial degradation of primary cutting fluid constituents

To examine the microbial degradation of the cutting fluid, 100 mg samples of three of its primary active ingredients—triethanolamine, tall oil, and organophosphate esters—were added separately to 150 mL aliquots of mineral medium without additional carbon sources. Nine such replicate samples were prepared for each ingredient. The composition of the mineral medium was 1.0 g/L K_2_HPO_4_, 1.0 g/L NaNO_3_, 0.5 g/L MgCl_2_ and 1.0 g/L MgSO_4_·7H_2_O in deionized water. The pH was adjusted to 8.5–9.0 using a 0.1 mol/L NaOH solution to mimic that of the cutting fluid used on-site. Samples of spoiled and sterile cutting fluid samples (2 mL each) were then inoculated into the test media and incubated at 25°C. The total organic carbon levels of each of these mixtures were tested after 0, 5, and 9 days of incubation, with three 10 mL samples tested from each mixture on a Multi N/C 3100 total organic carbon analyzer (Analytik Jena GmbH+Co., Jena, JN, Germany). The total volume was maintained at 150 mL with mineral medium. This approach facilitated precise quantification of which cutting fluid components were being actively metabolized by microorganisms.

## 3 Results and discussion

### 3.1 Microbial diversity in cutting fluid

**Figure 1:**
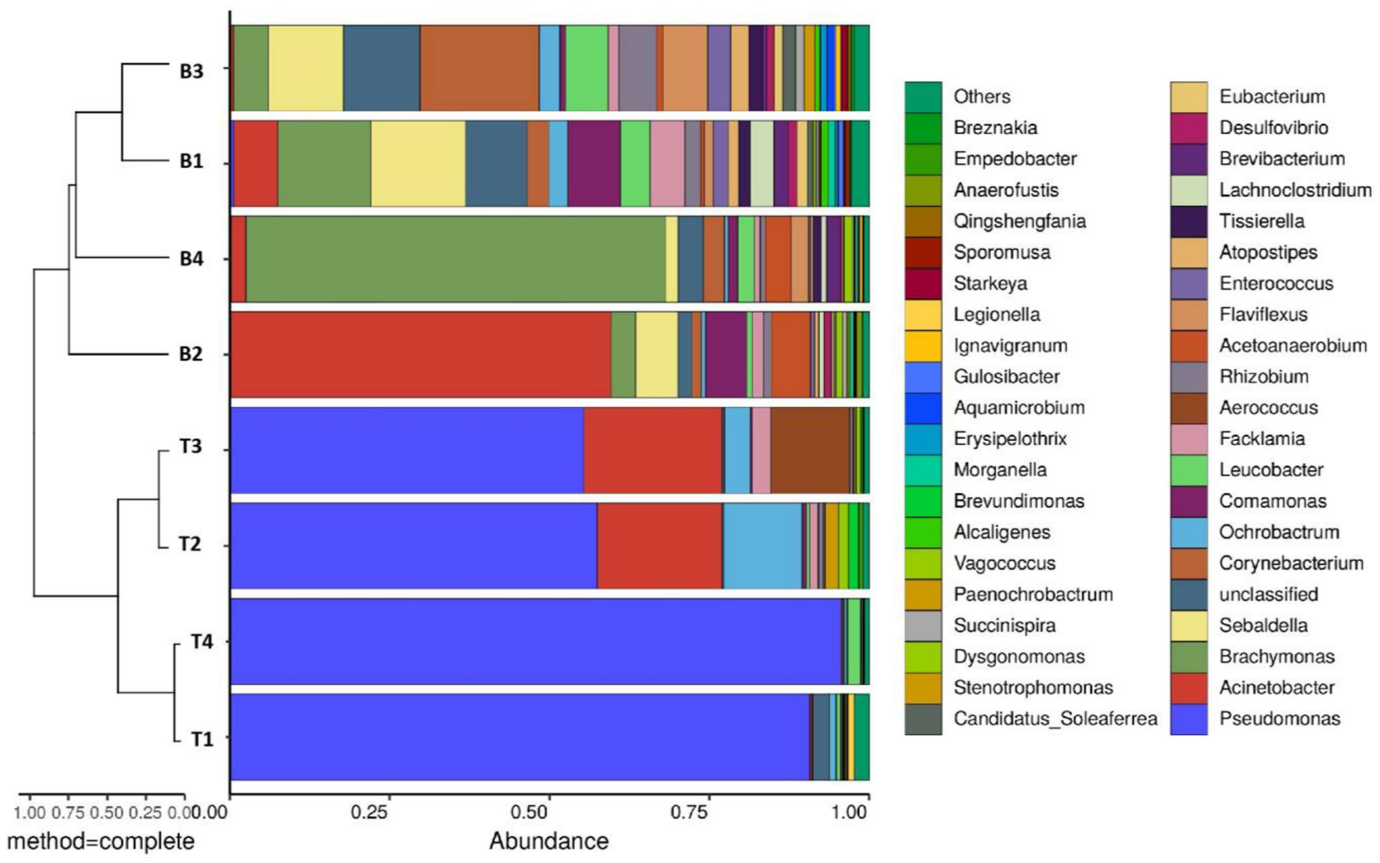
Combined analysis chart of genus-level sample clustering tree and bar chart.

Genus-level microbial community analysis revealed significant differences in the composition and relative abundances of the microorganisms retrieved from the surfaces and bottoms of the four machine tool tanks (Figure 1). The biodiversity was high in the B1 and B3 samples, and the relative abundances of the different microorganisms were relatively consistent. The microbial communities in the surface samples from the tanks of machines 1 and 4 were similar, being predominantly composed of *Pseudomonas.* Similarly, the microbial communities in the surface samples from the surface samples of machines 2 and 3 were also consistent, being predominantly composed of *Pseudomonas*, *Acinetobacter*, and *Ochrobactrum*. The bottom samples from all four machines mainly contained *Brachymonas*, *Sebaldella*, *Corynebacterium*, and *Comamonas*. Notably, the microbial communities in the surface samples had a high relative abundance of *Pseudomonas*, with percentages of 90.7, 57.5, 55.4, and 95.6% in samples 1–4, respectively. *Pseudomonas* is a facultative Gram-negative bacterium that thrives in aerobic conditions and can metabolize various carbon-, nitrogen-, and sulfur-containing compounds (Daniels et al., 2010; Babaei et al., 2014), thus facilitating the full degradation of various organic components (Caballero et al., 2005; Roca et al., 2009) and often leading to the deterioration of cutting fluid. The effective components in the cutting fluid were reduced by evaporation and splashing during the actual operational use of the cutting fluids. Microbial degradation represents a major factor that reduces the functional ingredients in cutting fluid (Rabenstein et al., 2009). Furthermore, the lower oxygen level at the bottom of the machine tool tank provides a favorable environment for the proliferation of anaerobic and facultative bacteria.

**Figure 2:**
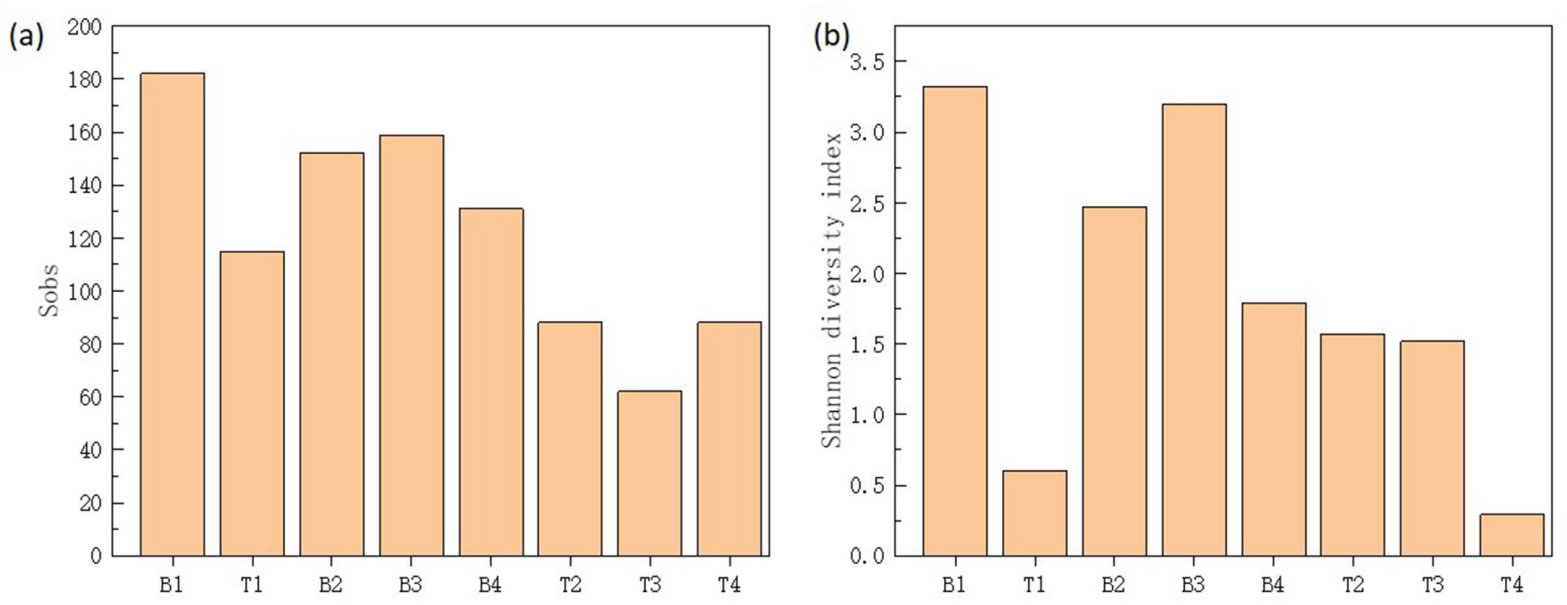
Sobs **(a)** and Shannon diversity index **(b)** of microbial communities in top (T1-4) and bottom (B1-4) cutting fluid samples taken from the four (1-4) machine tool tanks.

The Shannon index is used to measure the biodiversity of species. The higher the Shannon value is, the higher the microbial diversity will be (Lopes et al., 2024). The *α*-diversity of microbial communities in the four machine tool tanks were shown in Figure 2. The results showed that samples located bottom presented higher diversity and species richness in comparison with the samples located surface. The surface of the cutting fluid is usually in contact with air, and the oxygen content is relatively high. This oxygen-rich environment is conducive to the growth of aerobic microorganisms. For example, some bacteria of the genus *Pseudomonas* can utilize oxygen for respiration and decompose the organic components in the cutting fluid. However, at the bottom of the cutting fluid, oxygen diffuses relatively slowly, and it is easy to form an anoxic or even anaerobic environment. This provides living space for anaerobic microorganisms. These anaerobic microorganisms have unique metabolic pathways. As a result, aerobic, anaerobic and facultative anaerobic microorganisms coexist at the bottom, which increases the biodiversity. Based on the above results, it could be seen that sample B3 had a relatively high biodiversity and the relative abundances of each microorganism were relatively even. Therefore, sample B3 would be used as the source of strains and inoculated into the cutting fluid for experiments in the follow-up.

### 3.2 Microcosm experiment of cutting fluid microbial community

**Figure 3:**
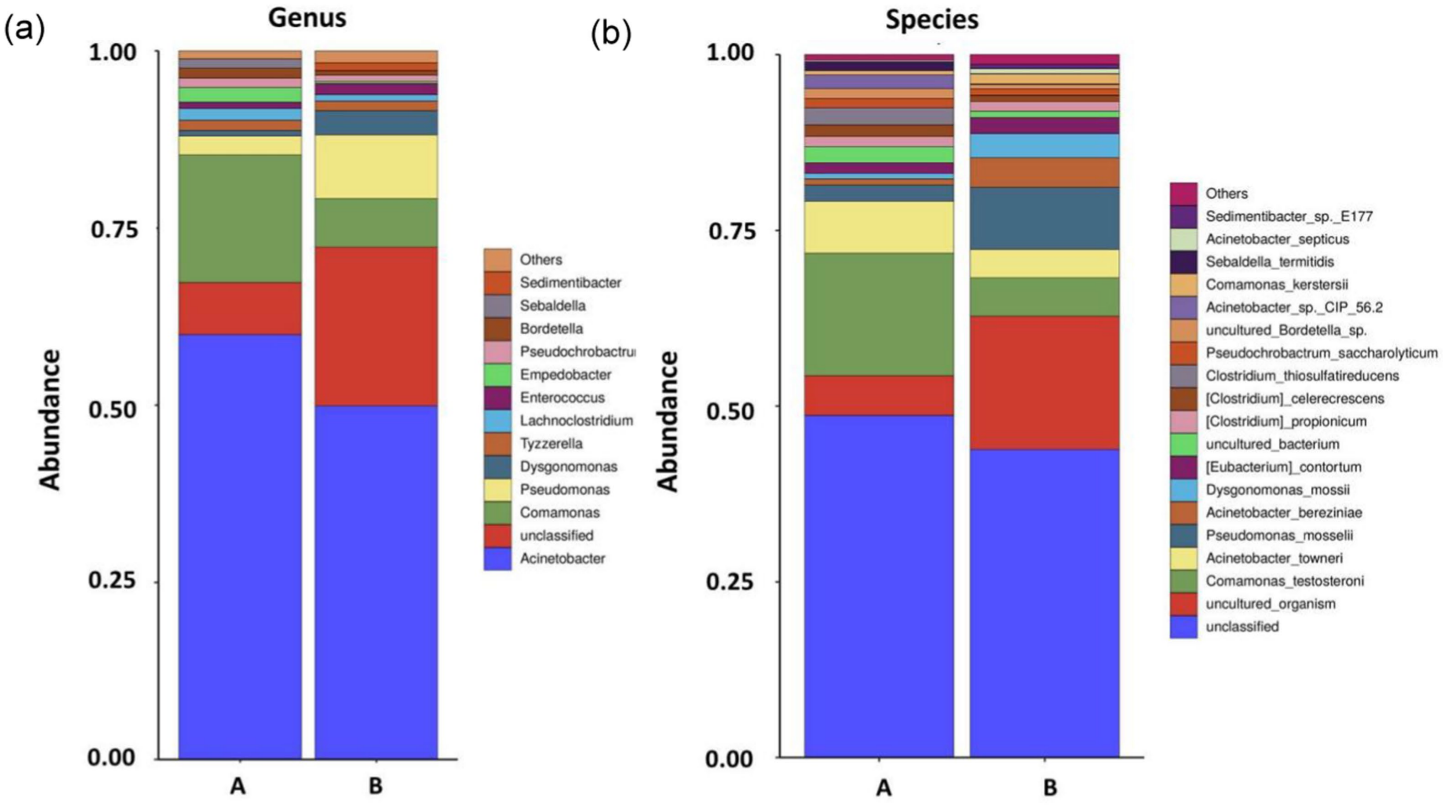
Microbial community structures and relative abundances at the conclusion of the enrichment cutting fluid culturing experiment at the genus **(A)** and species **(B)** levels (samples A and B are parallel samples).

The microbial community structure in the cutting fluid from the Test-SCF sample is shown in Figure 3. The microbial communities were predominantly composed of *Acinetobacter*, *Comamonas*, and *Pseudomonas* at percentages of 60.0, 18.0, and 2.7%, respectively in Sample A; and 49.9, 6.9, and 9.0%, respectively, in Sample B.

**Figure 4:**
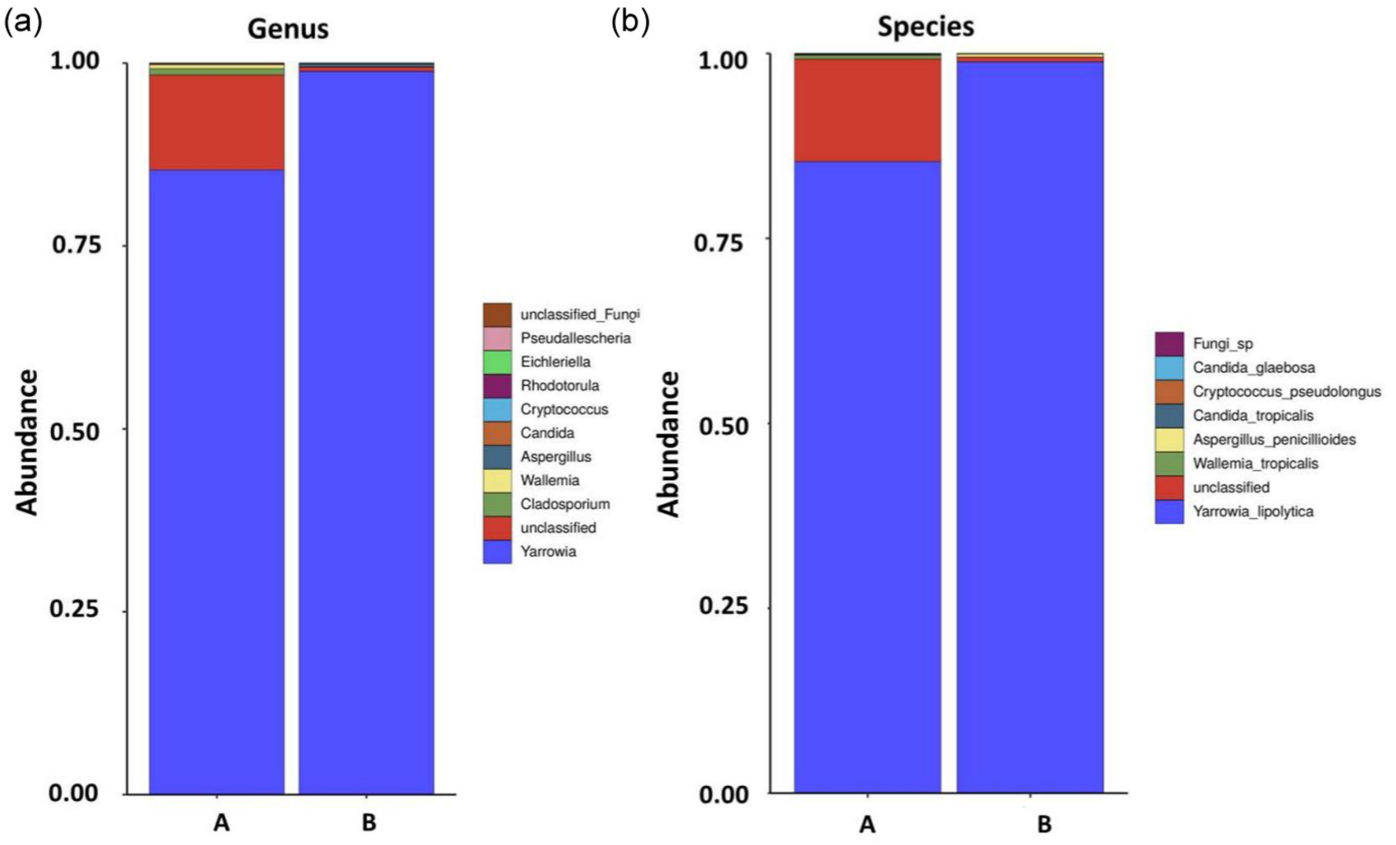
Fungal community structures and relative abundances in expanded experimental cutting fluids at the genus **(A)** and species **(B)** levels (samples A and B are parallel samples).

Genus- and species-level analyses of the fungal communities showed that, although there were fewer types of fungi than bacteria in the Test-SCF sample, their relative abundances were higher (Figure 4).

The experiment identified 85.4% *Yarrowia lipolytica* in sample A, and 98.9% in sample B, with 13.9% unclassified fungi in sample A, and < 1% each of *Wallemia_tropicalis*, *Candida_tropicalis*, *Cryptococcus*, *pseudolongus*, and *Candida_glaebosa. Yarrowia lipolytica* exhibited the highest relative abundance, indicating its dominance in the cutting fluid. This yeast is characterized by rapid reproduction, a robust colonization capability, a high survival rate, and resilience in low pH environments.

### 3.3 Microbial growth curve determination

**Figure 5:**
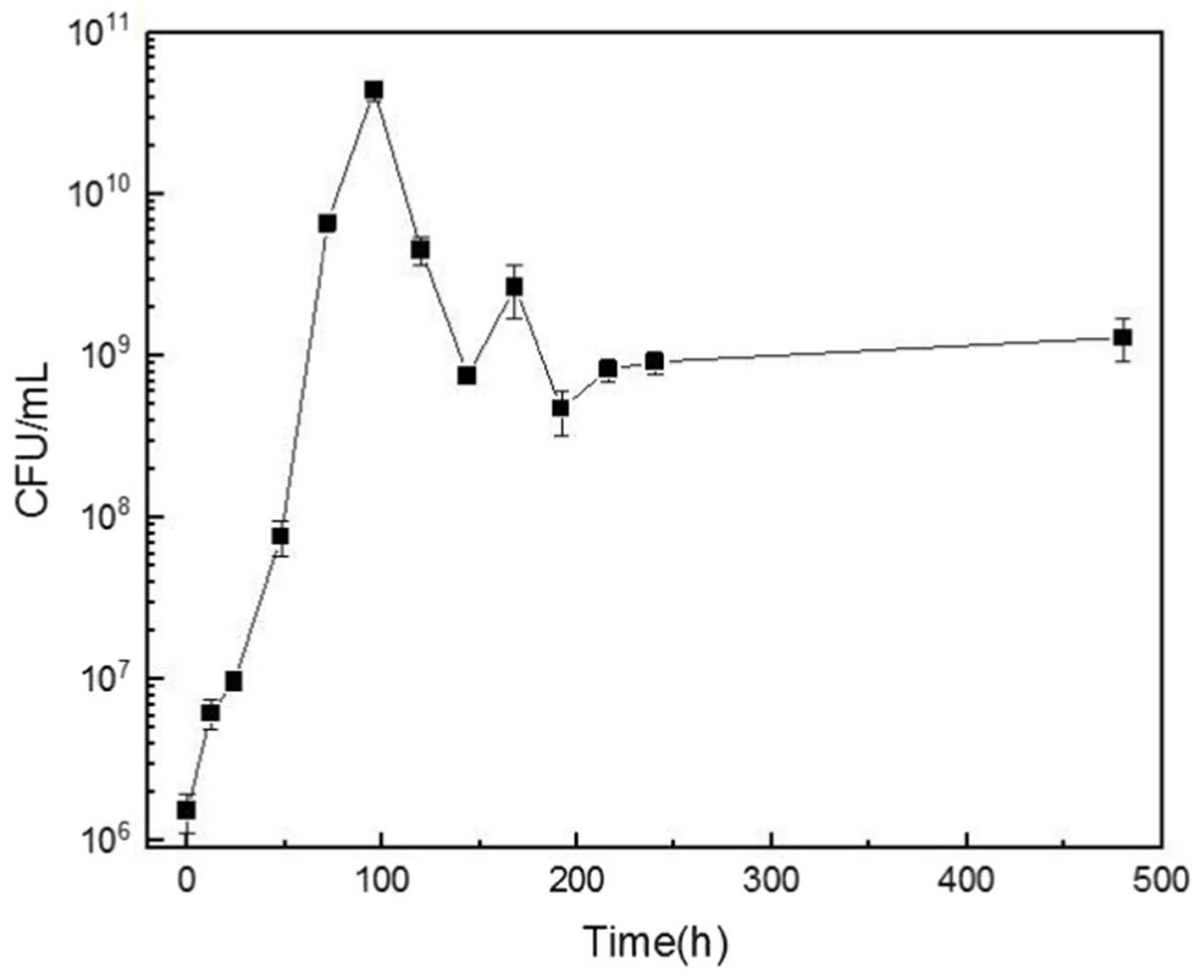
The variation curve of bacterial colony-forming units (CFU) per milliliter of cutting fluid over time.

The growth of microorganisms in the configured cutting fluid is presented in Figure 5. The graph indicates a slow microbial growth rate between 0 and 24 h, representing the lag phase. There was an exponential increase in biomass between 24 and 96 h, denoting rapid growth during the log phase. The biomass reached its peak at ~108 h, corresponding to the highest observed microbial concentration. After this point the biomass underwent a slight decline followed by a steady downward trend until 168 h, marking the onset of the death phase. Because of the high organic content in the cutting fluid that continuously supplied the necessary nutrients for microbial growth, the growth and death of the microorganisms eventually reached an equilibrium at a total count of ~10^11^ CFU/mL.

### 3.4 Impact of microorganisms on cutting fluid pH

The stability and corrosion-preventing physicochemical properties of cutting fluid are highly dependent on its pH, which is frequently used as a performance indicator in industrial testing. Cutting fluid pH should typically be maintained between 8.5–9.2 to ensure optimal performance.

**Figure 6:**
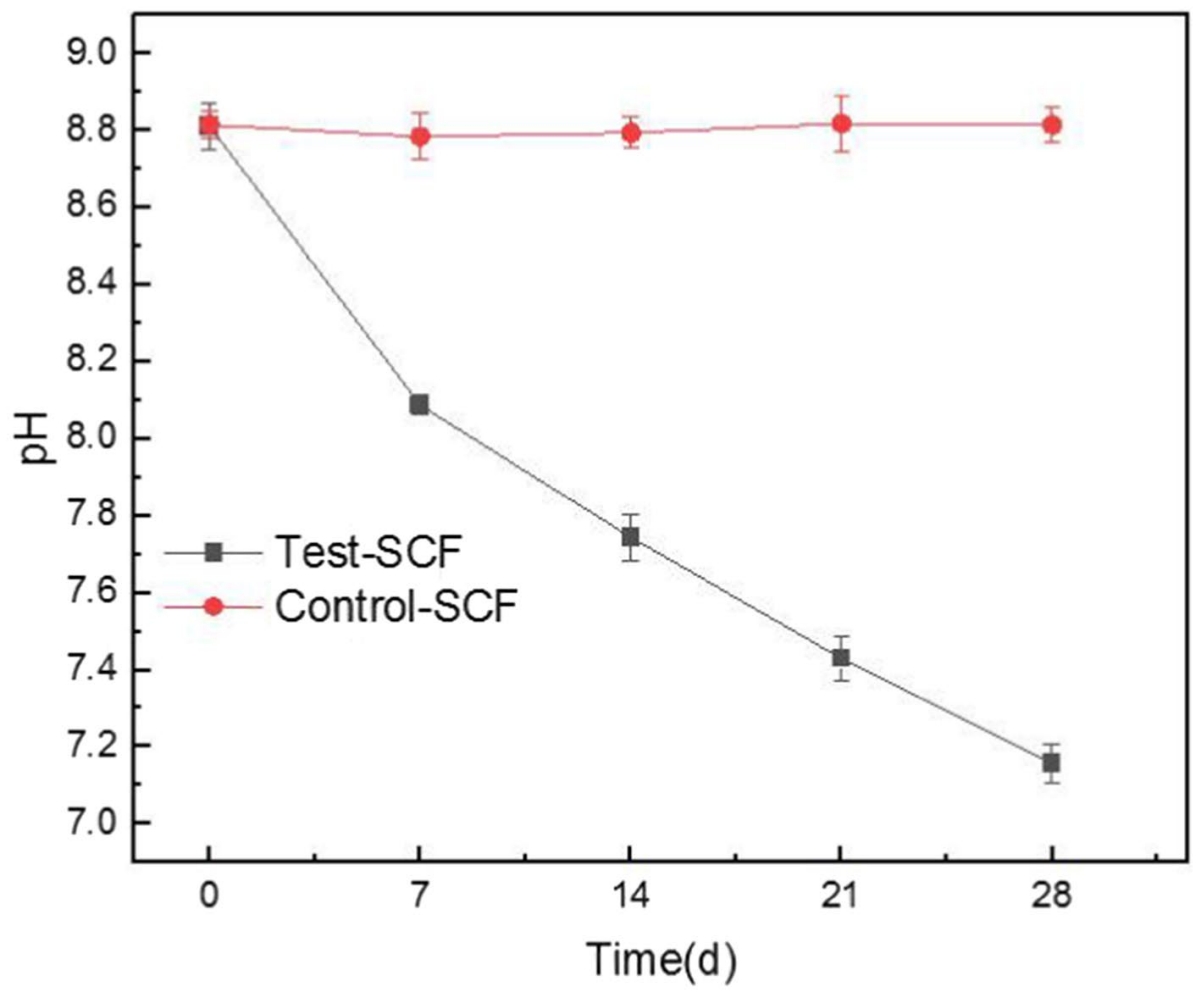
Trends in the pH values of the cutting fluids inoculated with bacteria (Test-SCF) and without bacteria (Control-SCF) over time.

Figure 6 displays the pH of cutting fluids over various time points, clearly showing that the pH of the cutting fluid sample that was inoculated with microorganisms decreased from an initial value of 8.81 to a final one of 7.15, whereas the pH of the uninoculated fluid did not change significantly. These results indicate that the presence of microorganisms significantly lowered the pH of the cutting fluid as the experiment progressed. The microbial growth curve indicates that microorganisms proliferate rapidly over time, which significantly impacts the pH of the cutting fluid. The microbial community analysis revealed the presence of various anaerobic bacteria, including the *Sebaldella* genus, at the bottom of the tool’s tank. These bacteria produce a range of organic acids such as acetic, lactic, and occasionally formic acid that reduce the pH of the cutting fluid (Li et al., 2023).

After being used for extended periods, cutting fluid harbors aerobic, facultative, and anaerobic bacteria. Aerobic bacteria can degrade the active ingredients of the fluid, leading to decreased performance. The metabolism of facultative and anaerobic bacteria produces organic acids, resulting in a lower pH (Ma et al., 2018). Moreover, *Y. lipolytica* produces significant quantities of citric acid that accumulate over time (Ledesma-Amaro et al., 2016). This bacterium also produces other acidic substances such as alpha-ketoglutaric acid and unsaturated fatty acids (Guo et al., 2016).

### 3.5 Impact of microorganisms on the corrosion-inhibiting properties of cutting fluid

**Figure 7:**
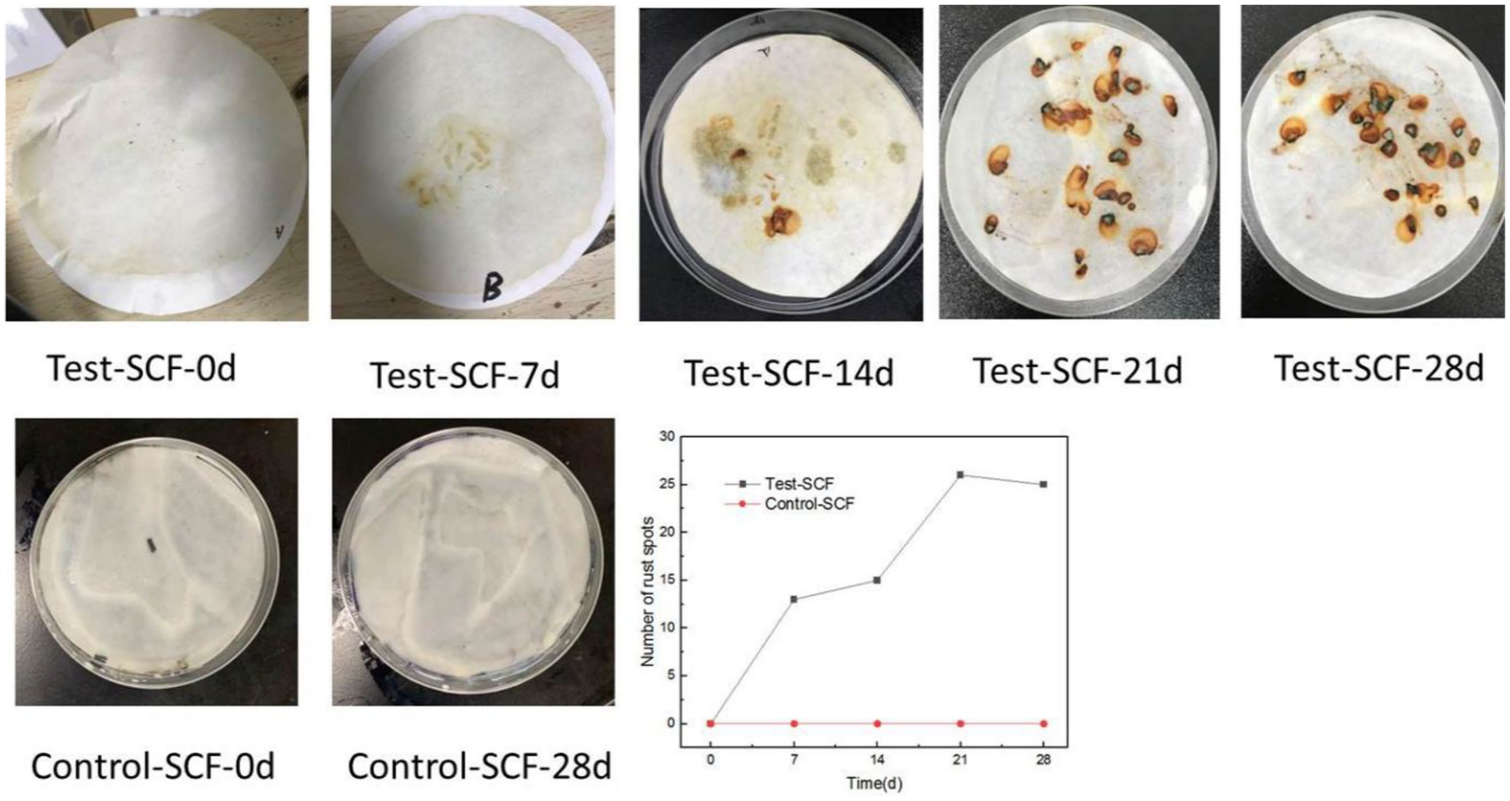
Iron filings experiment results and statistical map of the numbers of rust spots.

The iron filings experimental results are shown in Figure 7. No rust spots were observed in the Control-SCF sample after 28 days, indicating no impact on the corrosion inhibition properties of the sterile SCF. Similarly, no rust spots were observed when iron filings were added to the fresh Test-SCF sample, indicating that the newly-prepared fluid effectively inhibited their corrosion. However, as the experiment progressed, more rust spots appeared on the filter paper, indicating a decrease in the corrosion-inhibiting properties of the fluid. This deterioration is likely attributable to a rapid increase in the microbial population and a corresponding intensification in their metabolic activity that significantly reduced the corrosion-inhibiting properties of the fluid.

**Figure 8:**
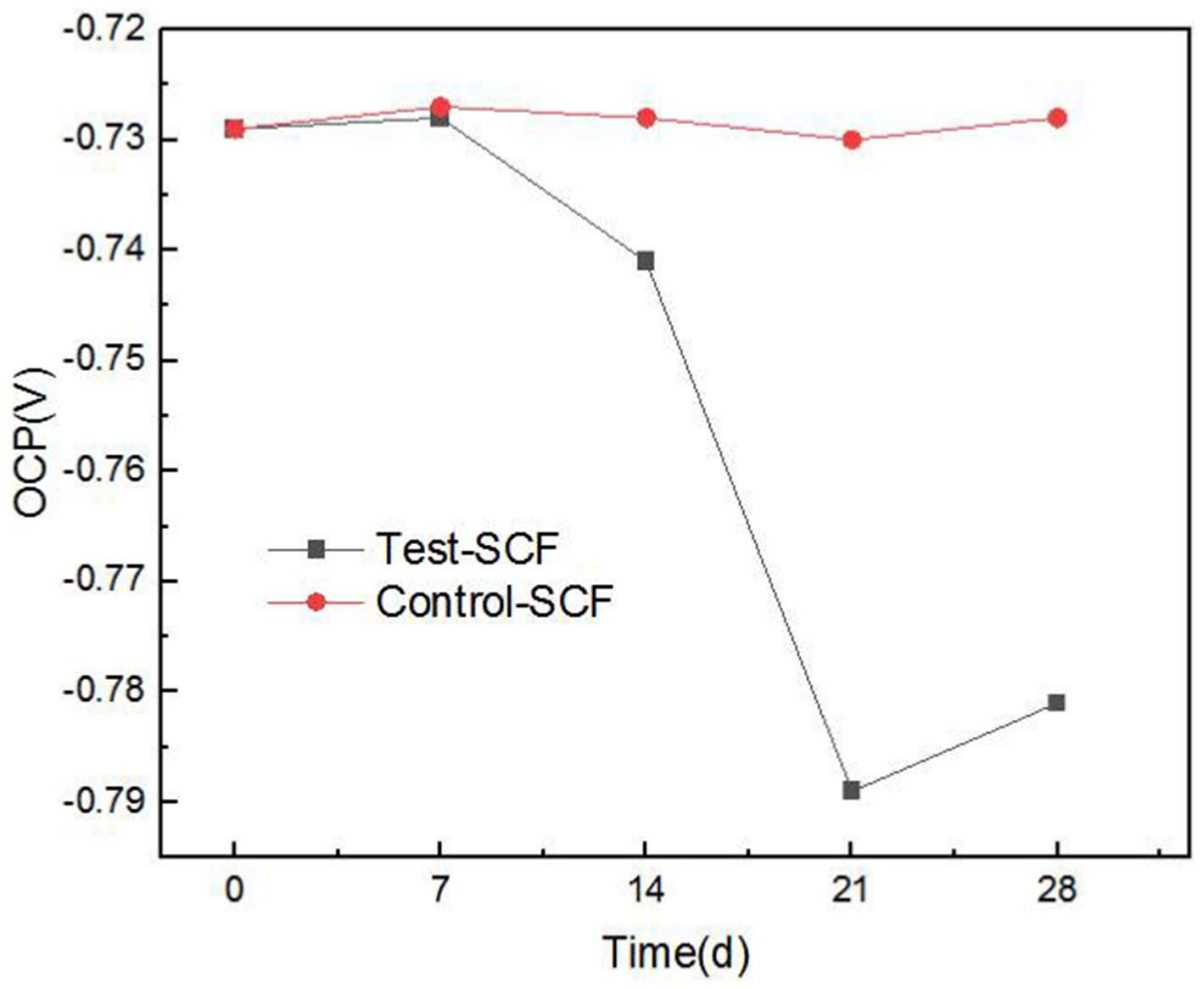
Open-circuit potential (OCP) trend in two cutting fluid samples over time.

As cutting fluid is primarily used to process aluminum alloys, this iron filings method does not comprehensively assess the impact of cutting fluid deterioration on its properties related to inhibiting the corrosion of aluminum alloy workpieces. Consequently, the effects of microbes on the ability of cutting fluid to inhibit corrosion were further investigated through electrochemical testing. Electrochemical tests were conducted using aluminum alloy samples that had been immersed in microorganism-inoculated cutting fluid for durations of 1, 7, 14, 21, and 28 days. Figure 8 presents the OCP of the aluminum alloy in the cutting fluid at various time points. Generally, a more negative OCP indicates that the alloy has a higher sensitivity to corrosion. The data revealed a noticeable downward trend in OCP, thereby suggesting that the sensitivity to corrosion of the aluminum alloy in the cutting fluid increased progressively. Cutting fluid includes corrosion inhibitors that are adsorbed onto metallic surfaces, exerting protective effects on the metal. Nevertheless, as the experiment progressed, the OCP of the aluminum alloy in the cutting fluid with microorganisms gradually declined. This was primarily attributed to the failure of the corrosion inhibitor to be adsorbed onto the surface of the aluminum alloy (Alobaid et al., 2024).

### 3.6 Impact of microorganisms on the lubricity of cutting fluid

**Figure 9:**
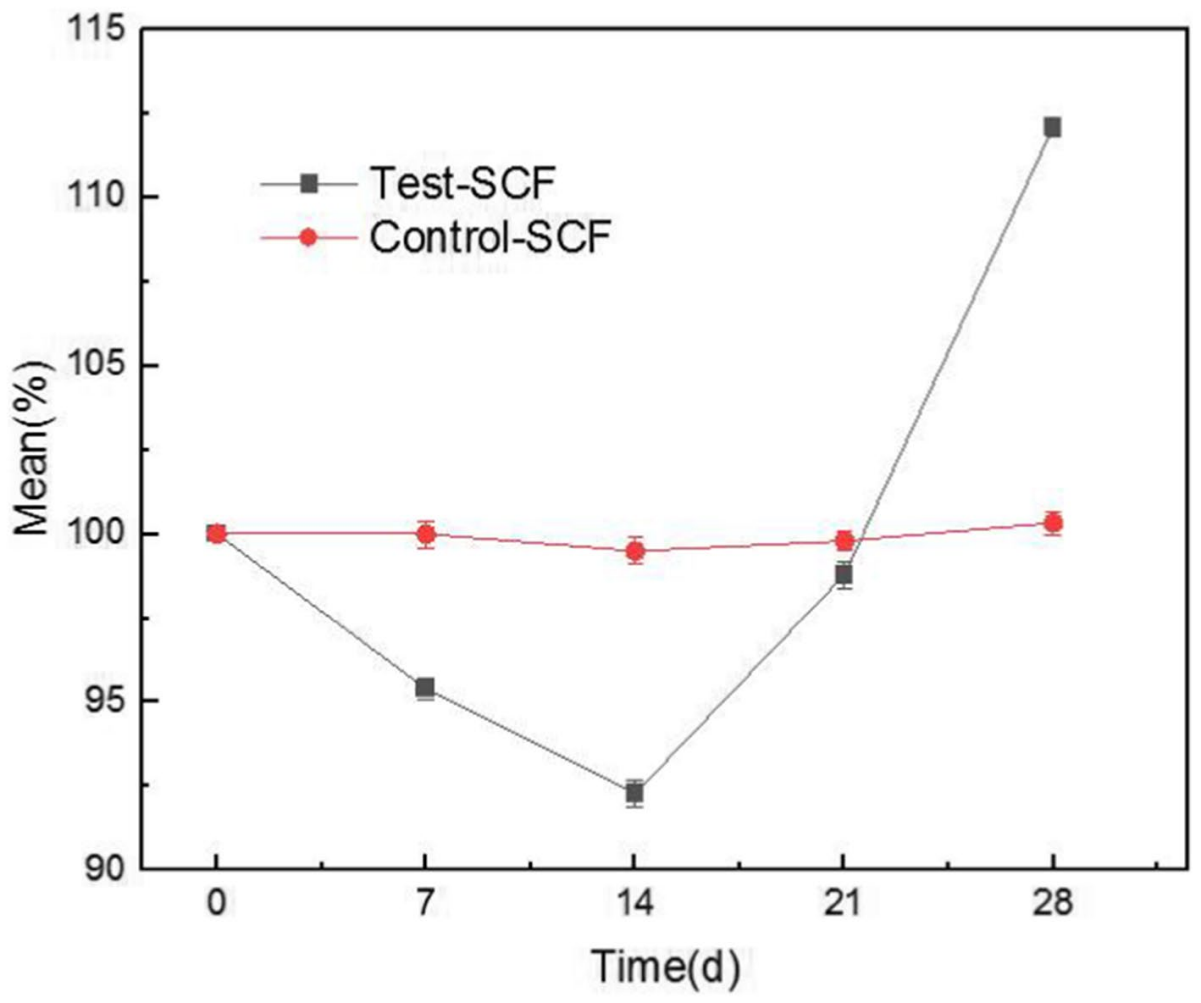
Trends in the tapping efficiency of two cutting fluid samples over time.

Figure 9 illustrates the tapping efficiency of the cutting fluid at different time points over the experiment. The results showed that the tapping torque value initially decreased gradually over time, before increasing again after 14 days. This suggests that in the early stage of the experiment, the presence of microorganisms slightly enhanced the lubricity of the cutting fluid (D’Addona et al., 2020). However, a biofilm was observed to have formed on the fluid’s surface. After stirring, clumpy substances appeared that resembled undissolved milk powder (Supplementary Figure S1). This observation was consistent with the characteristics of fungal growth, indicating that the number of fungi increased significantly. Fungal activity therefore likely became the determining factor, with *Y. lipolytica* representing the dominant fungus (Figure 4). In the cutting fluid environment with a high mineral oil content, *Y. lipolytica* follows a filamentous growth pattern (Ermakova and Morgunov, 1988). The growth and movement of fungal hyphae make the cutting fluid unable to form an effective lubricating film between the tool, chip, and machining surface (Elansky et al., 2022), resulting in reduced lubricity. Moreover, fungi produce various enzymes that can disrupt the active ingredients of cutting fluid, resulting in reduced lubricity (Buarque et al., 2023).

### 3.7 Impact of microorganisms on cutting fluid stability

**Figure 10:**
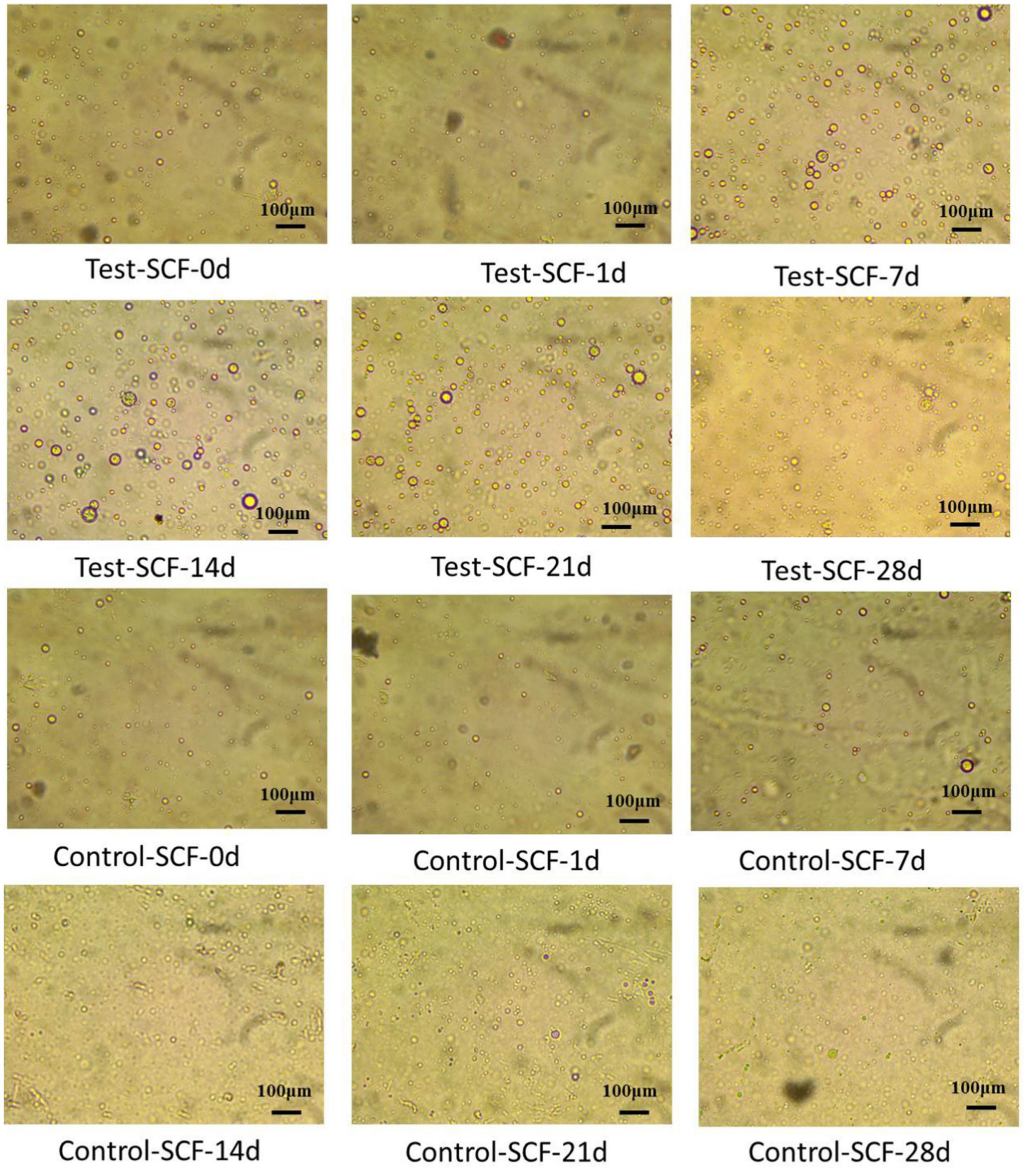
Droplet morphologies in two cutting fluid samples at various time points.

**Figure 11:**
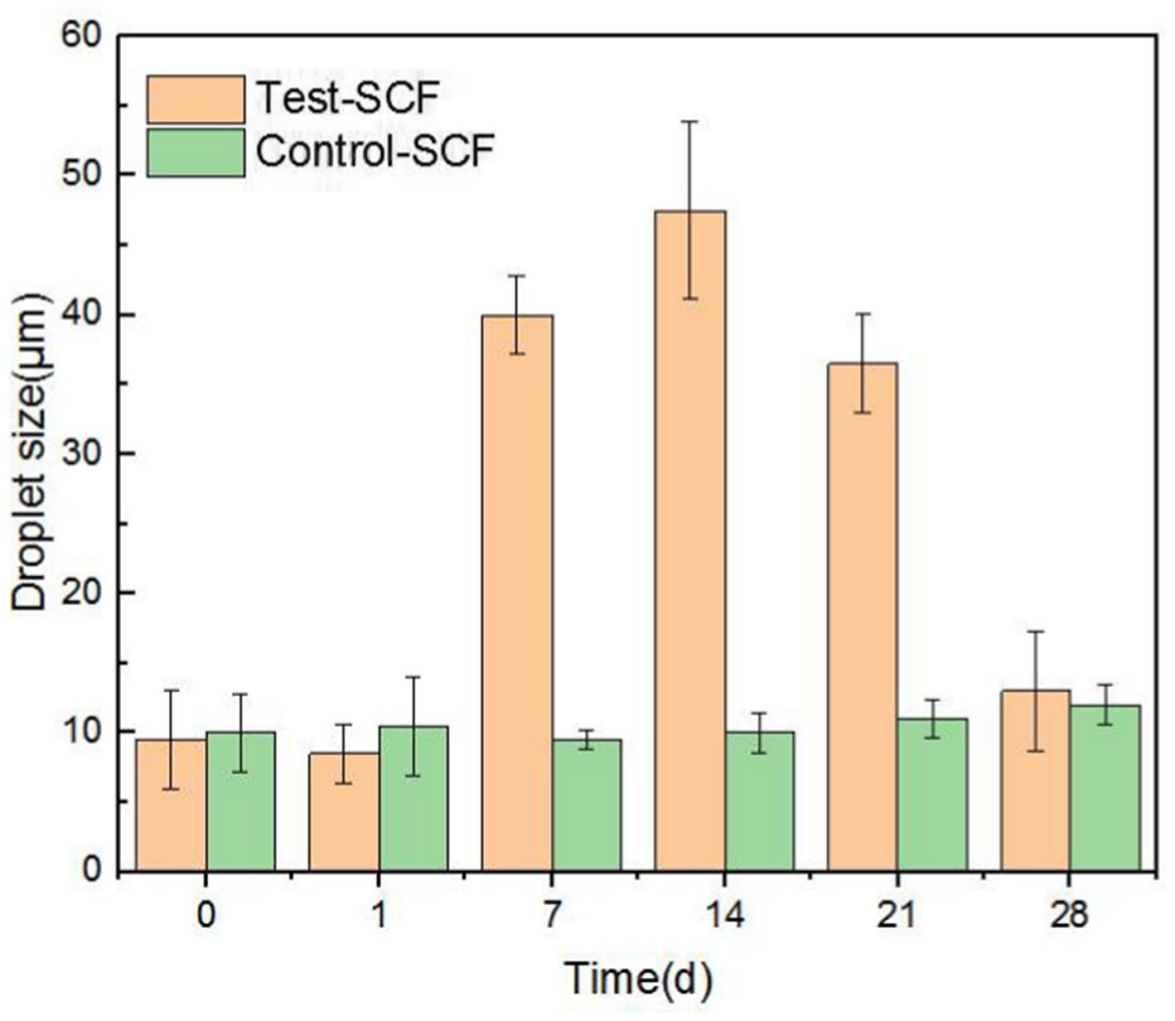
Diameters of randomly extracted droplets in two cutting fluid samples over time.

The impact of microorganisms on the stability of cutting fluid was assessed through droplet morphology and zeta potential. Figures 10, 11 show the morphology and diameter statistics of droplets in the cutting fluid at different time points over the experiment. The size of the oil droplets in the Test-SCF sample initially increased before increasing gradually after 14 days and stabilizing. Conversely, in the Control-SCF sample, the particle sizes of the oil droplets did not change appreciably, remaining stable at ~10 μm.

**Figure 12:**
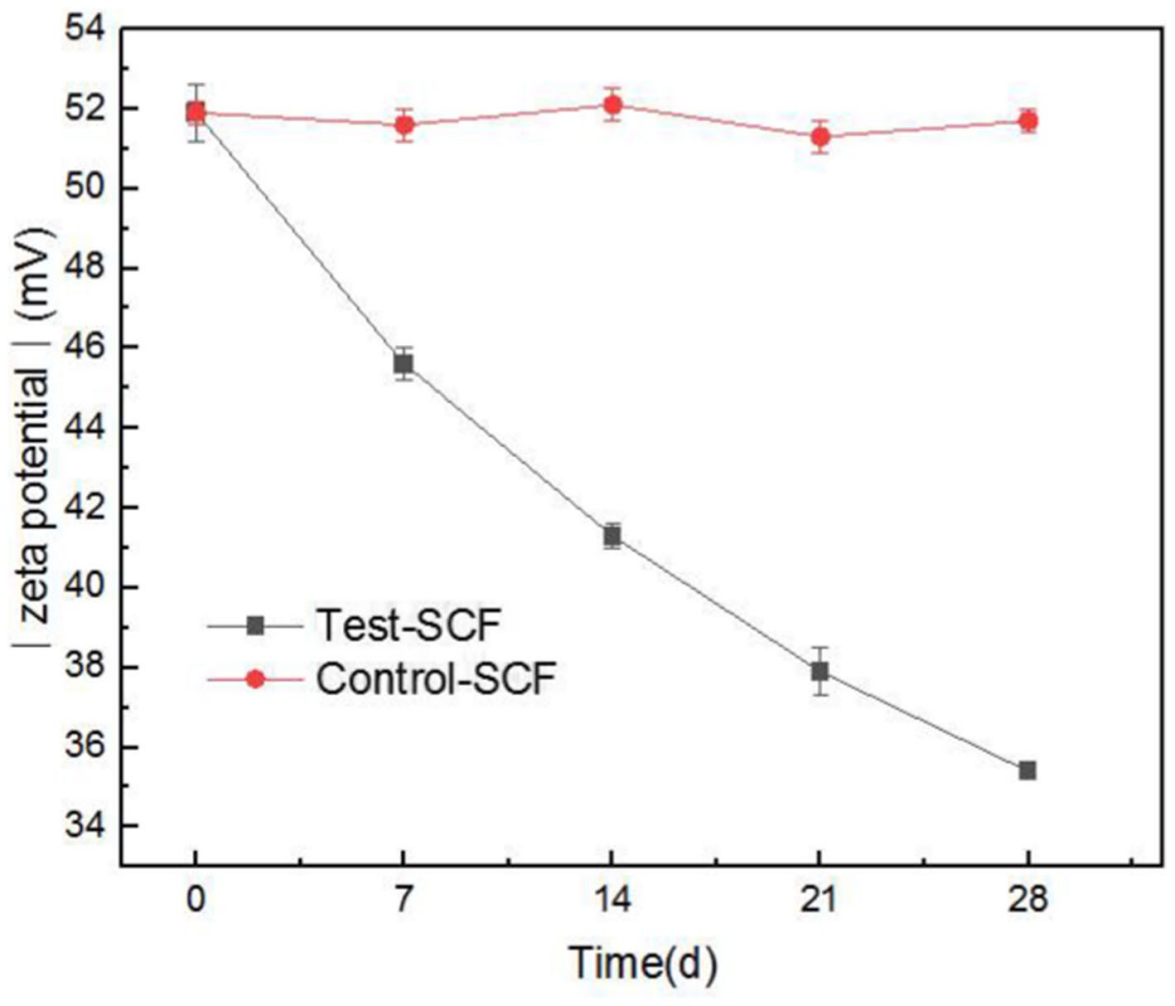
Absolute value of Zeta potential of two cutting fluid samples over time.

Zeta potential was used to show the stability of the cutting fluid system, with larger absolute values indicating a more stable system. Figure 12 shows the absolute value of the zeta potential of the freshly-prepared cutting fluid compared to the fluid that had progressed through 28 days of experimentation. The results indicated that the absolute zeta potential value decreased from 51.9 to 35.4 mV after 28 days. The significant reduction in the absolute value indicates a deterioration in the stability of the cutting fluid system.

This was primarily caused by the increased number of fungi after day 14 playing a dominant role. Fungal mycelia grew continuously in the cutting fluid, intertwining to form a network structure (Hotz et al., 2023). This led to the flocculation of oil droplets in the cutting fluid system, thereby reducing its stability. The effective components that provide lubrication also flocculated concurrently, resulting in the reduced lubricity observed in the tapping torque experiment results.

### 3.8 Impact of microorganisms on the reduction–oxidation potential of cutting fluid

**Figure 13:**
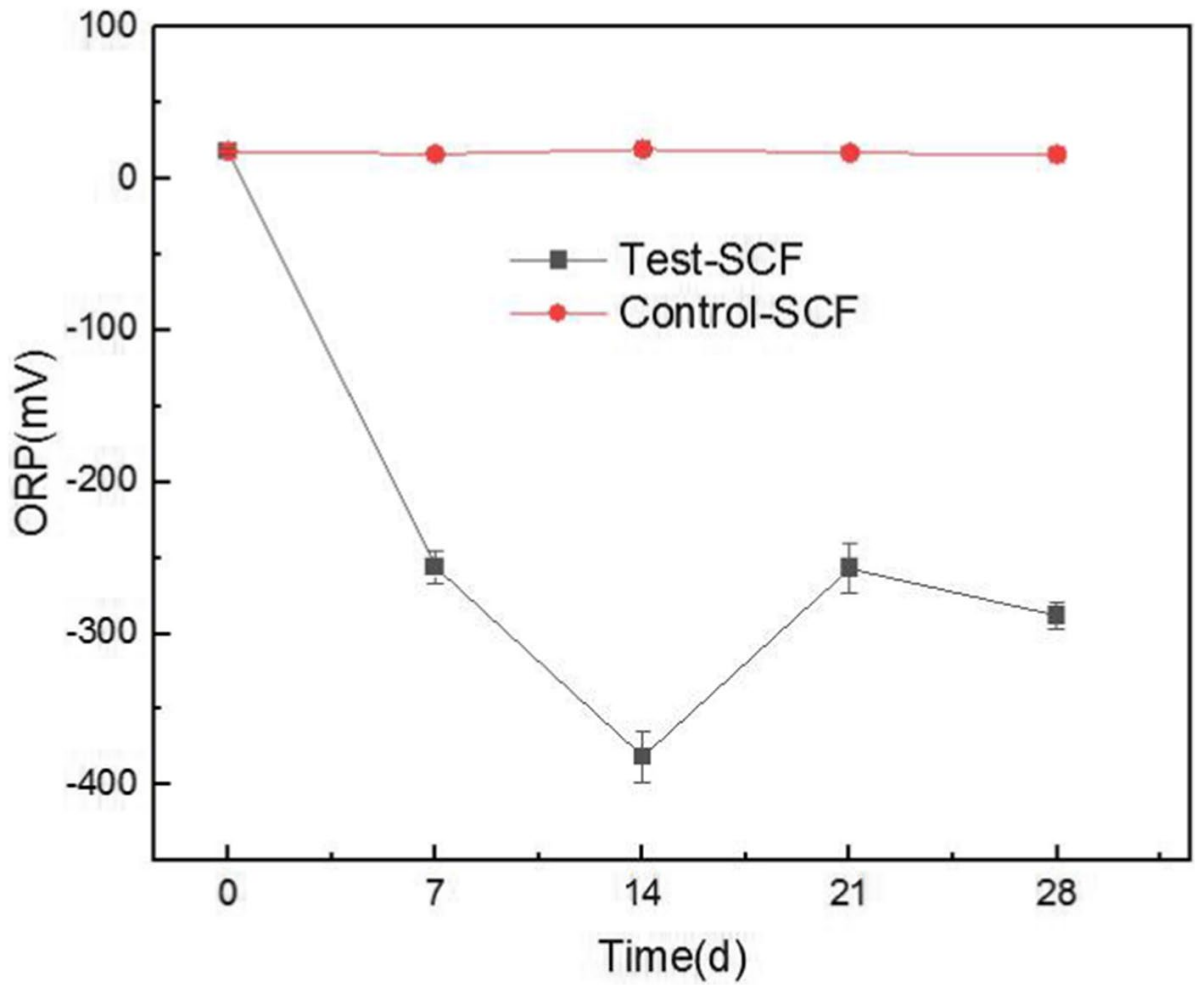
Redox potential (ORP) values of two cutting fluid samples over time.

ORP has rarely been used to assess the performance of cutting fluid, but our study innovatively used this property to analyze the cutting fluid samples. In most biological systems, aerobic cells exhibit high cell potentials, whereas anaerobic ones have low potentials. Enzymatic activity, cellular assimilation capabilities, and microbial growth are also influenced by ORP. The ORP of the cutting fluid gradually decreased over the test month and eventually stabilized at a relatively low value (Figure 13). This indicated that the number of anaerobic bacteria in the cutting fluid increased as the experiment progressed. For example, there was a significant presence of bacteria such as *S. termitidis* and *C. lubricantis*, and even the obligate anaerobe *C. celerecrescens* was detected. This demonstrated a clear correlation between ORP and the dominant microorganisms in the cutting fluid.

### 3.9 Mechanism analysis of the microbial effect on cutting fluid properties

In general, the growth and reproduction of microorganisms impacted the pH and corrosion inhibition capability of the cutting fluid in two ways. First, the microbial metabolic byproducts affected the pH and corrosion inhibition capability of the fluid. Second, the metabolic activity of the microorganisms themselves may have degraded the active ingredients of the cutting fluid.

Total organic carbon content was tested to investigate how microbial degradation affected the various components of industrial cutting fluid. Triethanolamine, which regulates pH, can cause pH imbalances in cutting fluid emulsions when degraded, thereby reducing performance (Pimenov et al., 2024). Tall oil, known for its excellent properties related to emulsification, lubrication, and corrosion inhibition, serves as a crucial emulsifying agent in cutting fluid (Kazeem et al., 2020). Organic phosphates, commonly added as corrosion inhibitors in cutting fluids for aluminum, help prevent corrosion during the machining process. These three substances were therefore selected to assess the effects of microbial degradation.

**Figure 14:**
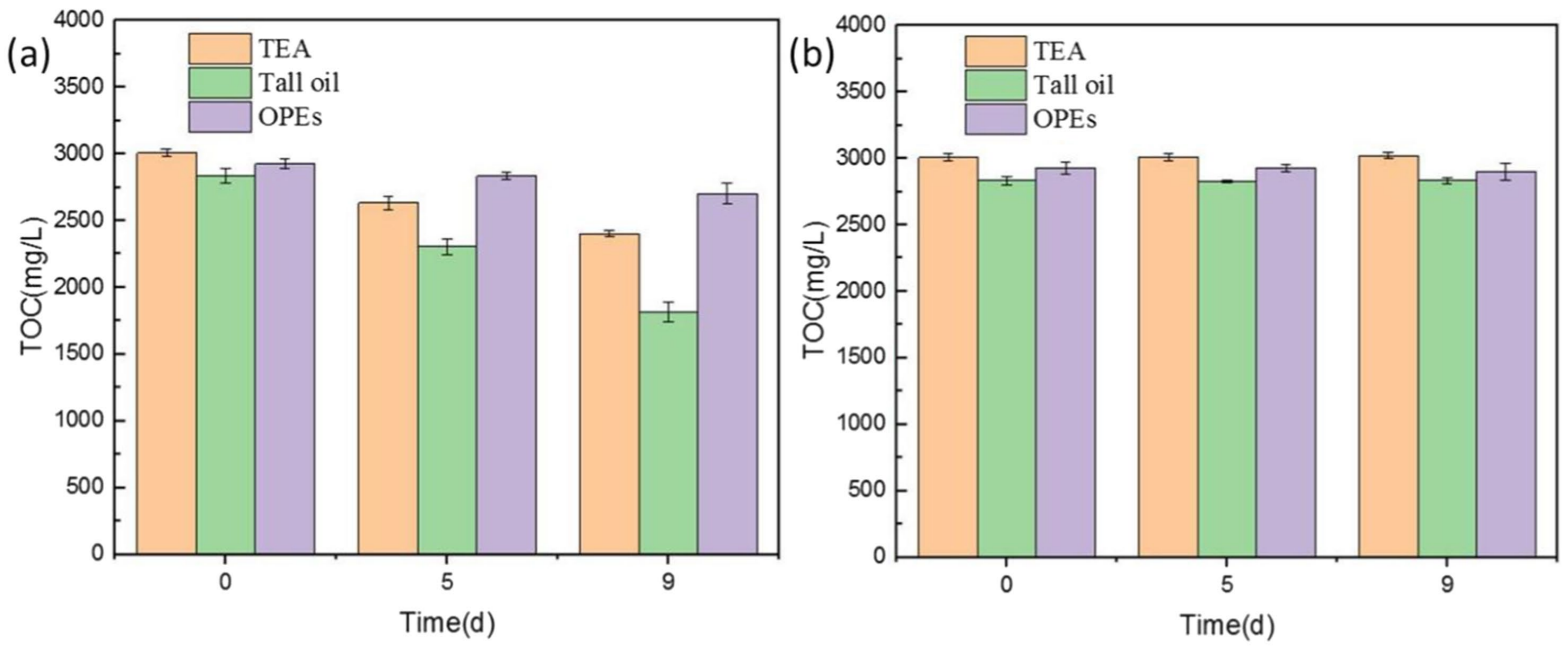
Total organic carbon (TOC) values of cutting fluid samples with **(A)** and without **(B)** microbial growth.

Figure 14 shows the effects of microbial degradation on these different components. Initially, the degradation rate of all three components was slow, owing to the lower microbial content and slower metabolism, thus resulting in a slower aerobic degradation rate. On day 5, during the logarithmic growth phase, the microbial population and metabolic activity increased substantially, accelerating the degradation of the organic components. By day 9, the degradation rates reached 26.82% for triethanolamine, 34.15% for tall oil, and 11.04% for organophosphate esters. The highest microbial degradation rates were observed for triethanolamine, followed by tall oil, with the organophosphate esters showing the lowest rates. Although their rate of microbial degradation was relatively slow, the organophosphate esters were nevertheless degraded. This indicates that, during metabolism, microorganisms can use corrosion-inhibiting organophosphate esters as a nutrient source, thus reducing the corrosion-inhibition properties of cutting fluid. Microorganisms also degraded triethanolamine, a pH regulator, ultimately leading to lowered pH in the cutting fluid.

## 4 Conclusion

The microbial compositions of the spoiled cutting fluid samples varied significantly between those taken from the surface and the bottom of the onsite instrument’s liquid tank. The surface layer predominantly contained aerobic bacteria such as *Pseudomonas*, *Acinetobacter*, and *Ochrobactrum*. Conversely, the bottom layer was primarily composed of anaerobic and facultative bacteria such as *Brachymonas*, *Sebaldella*, *Corynebacterium*, and *Comamonas*. The results of laboratory microbial culturing experiments showed that the fungal communities in these samples mainly consisted of the yeast *Yarrowia lipolytica*. Microbial metabolic activity inhibited the formation of passivation films on the aluminum alloys, through the secretion of various organic acids. These activities also consumed the primary active ingredients of the cutting fluid, leading to a significant decrease in its pH and ability to inhibit corrosion. The presence of microorganisms slightly enhanced the lubricity of the cutting fluid; however, in the later stages of the experiment, an increase in fungi, as well as the corresponding sweeping and bridging actions of the fungal hyphae, caused the active ingredients in the cutting fluid system to flocculate, reducing both its lubricity and stability. The results of this study showed that microorganisms impact all properties of industrial cutting fluid, with the most pronounced effects being related to corrosion inhibition, pH, and stability. The experimental conditions were restricted to a laboratory environment, so there may be many other potential microbial species and influencing factors related to this phenomenon that have remained unexplored. A deeper understanding of the impact of microorganisms on the deterioration of cutting fluid performance plays a key role in improving the quality, efficiency, and sustainability of the cutting process. It will also hopefully lay a theoretical foundation for the future development and application of new cutting fluids.

## Data Availability

The data presented in the study are deposited in the National Center for Biotechnology Information (NCBI) repository, accession numbers SRP554559 and PRJNA1204126.
