# Supplementary figures and images for: Microbial proliferation deteriorates the corrosion inhibition capability, lubricity, and stability of cutting fluid
Source: Front Microbiol. 2025 Feb 11;16:1522265. doi: 10.3389/fmicb.2025.1522265 (PMC11850348; doi:10.3389/fmicb.2025.1522265)

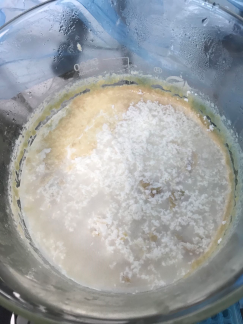

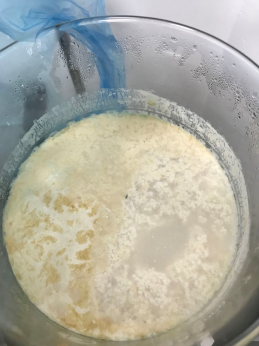


(b)

(a)

Fig. S1 The biofilm on the surface of cutting fluid after 21d (a) and 28d (b) experiment

Supplement: Supplementary file 1 [file Data_Sheet_1.docx]
